# 2-year outcome of juvenile idiopathic arthritis in current daily practice: what can we tell our patients?

**DOI:** 10.1186/1546-0096-9-S1-P153

**Published:** 2011-09-14

**Authors:** Janneke Anink, Koert M Dolman, Merlijn J van den Berg, Mira van Veenendaal, Taco W Kuijpers, Marion AJ van Rossum

**Affiliations:** 1 Pediatric Rheumatology, Emma Children’s Hospital, Academic Medical Center, Amsterdam, The Netherlands; 2 Department Pediatric Rheumatology, Reade location Jan van Breemen,Amsterdam, The Netherlands; 3 Department of Pediatrics, St Lucas Andreas Hospital, Amsterdam, The Netherlands

## Background

As treatment options in juvenile idiopathic arthritis (JIA) are quickly developing, limited data exist that appear fully applicable for informing our newly diagnosed JIA patients about the expected course of their disease

## Aim

To evaluate disease course and outcome of patients in the first two years after diagnosis JIA when treated according to local standard of clinical care.

## Methods

We performed a retrospective inception cohort study of children with JIA, diagnosed between 2003 and 2007, treated in referral centers in Amsterdam. Disease status was determined for every outpatient clinic visit. Data regarding medication, functional outcome and radiography were recorded.

## Results

149 consecutive newly diagnosed JIA patients were included. Median age at diagnosis was 11.8 years with a median follow up of 33 months. DMARDs were used in 95% of the patients including methotrexate in 85%, sulfasalazine in 41% and biologicals in 20%. DMARDs were started within median one month after diagnosis. During follow-up 77% of patients achieved a total of 244 episodes of inactive disease (ID). ID was reached after median 10 months. After 2 years a median CHAQ score of 0.6 was reported. Radiological joint damage occurred at some point in 12% of patients: 3% showed damage at diagnosis and another 9% developed joint damage within a median of 20 months after their first clinic visit.

## Conclusions

With current management strategies in daily clinical practice, 77% of newly diagnosed JIA patients achieve a first episode of inactive disease within a median of 10 months. After 2 years, patients report a moderate functional disability and more than 10% show radiological evidence of joint damage.

